# Preoperative short-term radiation therapy (25 Gy, 2.5 Gy twice daily) for primary resectable rectal cancer (phase II)

**DOI:** 10.1038/sj.bjc.6602485

**Published:** 2005-03-22

**Authors:** J Widder, F Herbst, W Dobrowsky, R Schmid, B Pokrajac, B Jech, C Chiari, A Stift, A Maier, J Karner-Hanusch, B Teleky, F Wrba, R Jakesz, R Poetter

**Affiliations:** 1 Department of Radiotherapy and Radiobiology, Medical University of Vienna, Waehringer Guertel 18-20, A-1090 Vienna, Austria; 2 Department of Surgery, Medical University of Vienna, Waehringer Guertel 18-20, A-1090 Vienna, Austria; 3 Northern Centre of Cancer Treatment, Newcastle General Hospital, Westgate Road, Newcastle/Tyne NE4 6BE, UK; 4 Department of Radiology, Medical University of Vienna, Waehringer Guertel 18-20, A-1090 Vienna, Austria; 5 Department of Clinical Pathology, Medical University of Vienna, Waehringer Guertel 18-20, A-1090 Vienna, Austria

**Keywords:** bowel function, rectal cancer, rectal carcinoma, short-term preoperative radiotherapy, surgery

## Abstract

To evaluate the feasibility, effectiveness, and long-term bowel function of preoperative hyperfractionated accelerated radiotherapy in primary resectable rectal cancer. A total of 184 consecutive patients (median age 65 years, male : female=2 : 1) with clinical T3Nx rectal adenocarcinoma received preoperative pelvic radiation therapy with single fractions of 2.5 Gy twice daily (interval 6 h between fractions) to a total dose of 25 Gy within 1 week. Surgery was conducted the following week. Postoperative histology revealed UICC stage I in 33%, stage II in 26%, stage III in 34%, and stage IV in 7% of the patients. Median follow-up was 43 months (53 months for surviving patients). The actuarial 4-year-local-recurrence rate was 2.1%, overall recurrence 23%. Disease-specific and disease-free survivals at 4 years (excluding stage IV) were 82 and 69%, respectively. Overall survival for 4 years was 68%. Postoperative mortality was 0.5% (one patient), early anastomotic leakage occurred in 11.4%, and anastomotic stenosis requiring treatment in 6%, of 132 patients with primary anastomosis. Seven of 184 patients (3.8%) died of abdominal complications, all within the first year. Bowel function was satisfactory after more than 5 years. Local control in primarily resectable rectal cancer after 10 × 2.5 Gy is excellent, warranting further evaluation of this treatment.

Local tumour control remains an important aim in the treatment of rectal cancer because of devastating morbidity and unsatisfactory treatment options for local recurrence (Esnaola *et al*, 2002). Surgical advances in recent years have led to a significant reduction of local recurrences particularly due to standardised introduction of total mesorectal excision (TME) (MacFarlane *et al*, 1993). Independently, neo-adjuvant or adjuvant radiotherapy reduces the incidence of local relapse by 50% as shown by meta-analyses (Camma *et al*, 2000; Colorectal Cancer Collaborative Group, 2001). The positive effect of radiation is maintained even with quality-controlled TME surgery (Kapiteijn *et al*, 2001).

Preoperative radiation therapy using 25 Gy with daily fractions of 5 Gy, administered within 1 week with surgery performed during the following week, is a widely tested treatment regimen. Its efficacy regarding prevention of local recurrence has been documented in several thousand patients (Glimelius, 2002) and it has been shown to increase the overall survival by 10% in one large trial (Swedish Rectal Cancer Trial, 1997). Nevertheless, concern remains within the oncology community regarding the daily 5 Gy dose per fraction. We have therefore introduced a modification of the 5 × 5 Gy treatment regimen at our department in 1994. The total dose of 25 Gy is given by single fractions of 2.5 Gy delivered twice daily with an interfraction interval of at least 6 h, whereby the overall treatment time of 1 week is maintained. Sufficient recovery of normal tissue after 6 h has been shown to occur in several clinical situations so that late reactions are not expected to increase compared to conventional fractionation (Dische *et al*, 1997). Thus, the low total dose of 25 Gy is predicted to translate into a low probability of negative late adverse effects (see Appendix A).

We report the efficacy, toxicity, and long-term functional results of the first 184 consecutive patients treated between 1994 and 2000 with this new regimen at the Department of Radiotherapy and Radiobiology at Medical University of Vienna General Hospital.

## PATIENTS AND METHODS

Patients with histologically proven adenocarcinoma of the rectum without evidence of distant metastases were eligible, if transmural extension was to be expected upon digital examination, rectoscopy, and pelvic computed tomography (CT) scan. Pelvic MRI was not used for staging. A complete resection of all tumour tissue, either by low anterior resection (LAR) with primary anastomosis or by abdominoperineal resection (APR), was to be judged feasible by an experienced rectal surgeon. Patients with large T3 tumours, where a radical resection appeared to be uncertain due to adherence to the pelvic side-wall, to the presacral fascia, or to adjacent organs, or patients with obvious T4 tumours received long-term preoperative radiochemotherapy with downsizing intent and are not included in this analysis. Patients with suspected T1/T2 tumours upon clinical and radiological evaluation without evidence of positive lymph nodes were not referred for radiotherapy, as were patients who had undergone previous pelvic radiotherapy for other reasons. Synchronous tumours in the colon were ruled out by colonoscopy or by barium enema, distant metastases by CT scans of the chest and abdomen.

Patients provided written informed consent upon participation and the protocol was approved by the institutional review board of Vienna Medical University.

Follow-up was every 3 months during the first year post-treatment, every 6 months during the second and third years, and yearly thereafter. Clinical evaluation was supported by checking tumour markers CEA and CA 19-9, abdominal-pelvic CT scans or MRI, and chest X-ray as appropriate. In cases of rising tumour markers, a complete screening for recurrence was performed. Suspicious intrapelvic tissue was followed by CT or MRI and histological exploration was performed in cases of remaining uncertainty. Bowel function was assessed in 2003 and 2004 for patients treated in 1994–1998.

### Radiotherapy

The clinical target volume comprised the tumour, the mesorectal tissue including perirectal and presacral nodes, and internal iliac lymph nodes (Widder *et al*, 2000). The caudal boundary of the clinical target volume was at 5 cm caudal to the macroscopic tumour as assessed by rectoscopy, digital rectal, and fluoroscopic investigation using a small rectal barium enema at simulation. Therefore, the anus was included only in very low tumours where a safety margin could not otherwise be obtained. The perineum was not included in the target volume even if an APR was planned. Patients were simulated in prone position with full bladder and barium enema of the small intestine. They were treated using a four-field box technique from 1994 to early 1997, and with three fields (same target volume) with a posterior and two lateral opposing wedged fields thereafter. Individual shielding was obligatory by individual shielding blocks or multileaf collimation.

Single doses per fraction were 2.5 Gy calculated at the ICRU (1993) point. Fractions were delivered with 6 or 10 MeV photons for the posterior portal, and with 25 MeV for the anterior (if used) and the lateral portals. Wedges to increase dose homogeneity were calculated on the basis of at least three relevant CT sections. Two fractions with intervals between fractions of at least 6 h were delivered every day from Monday to Friday to result in a total dose of 25 Gy within 1 week.

### Surgery

Surgery was planned within 1 week after radiotherapy. Employing a midline laparotomy, the splenic flexure was mobilised routinely with high ligation of inferior mesenteric vein and artery. Following sharp perimesorectal dissection, the rectum was divided at least 2 cm and the mesorectum at least 5 cm below the tumour. This resulted in a TME operation for all tumours located in the middle and lower thirds of the rectum, respectively. Only for lesions in the upper third a partial mesorectal excision was employed. Abdominal dissection was identical for patients undergoing an APR. More than 90% of the anastomoses were stapled; the remainders were hand-sewn. Short colon-J-pouches were added at the discretion of the operating surgeon, as were diverting ostomies.

### Bowel function

Bowel function was assessed at the last follow-up later than 5 years after treatment. Assessment was conducted by the doctor in the outpatient clinic or by telephone by directly inquiring the following items: (1) frequency of bowel emptying per day: one or less/two to three/more than three. (2) Clustering: one portion/two to three portions/more than three portions. (3) Ability to delay defecation upon urge: more than 15 min/less than 15 min. (4) Ability to distinguish between stool and gas: yes/no. (5) Experiencing tenesms: no/yes. (6) Wearing pads: no/yes. (7) Experiencing pelvic pain: no/yes. (8) Quality of life altered because of bowel function: no/yes/significantly.

### Statistics

Survival and recurrence data were calculated by the Kaplan–Meier method using SPSS statistical software (SPSS, Chicago, version 8.0 for Windows). Recurrence, overall, disease-specific, and disease-free survivals were assessed for all patients and for patients without primary metastases (stages I–III), respectively. Results for UICC stages were compared using the log-rank statistic. Of note, local recurrence was assessed by counting any local recurrence as event, regardless of whether this occurred as first recurrence or after metastasis. Patients who had not undergone a macroscopically complete tumour resection (four patients) were not assessable for local failure because they were never rendered free of local tumour. Nevertheless, actuarial local control for all patients including those incompletely resected is also reported.

## RESULTS

### Patients

From February 1994 to December 2000, 184 patients with histologically proven adenocarcinoma of the rectum with clinical or radiological (CT) signs of transmural extension or with suspicious pelvic lymph nodes were referred for radiotherapy. This report includes the results of a consecutive cohort of patients without proven distant metastases prior to treatment, who seemed primarily amenable to curative oncological surgery.

In all, 65% of the patients were men, 35% women; median age was 65 years (range 32–89). Postoperative UICC and pTNM stages, and types of surgery are displayed in Table 1. A total of 61% of the tumours were located in the lower third of the rectum, 29% in the middle third, and 10% in the upper third.

### Radiotherapy and surgery

An amount of 25 Gy was given with single doses of 2.5 Gy twice daily with interfraction intervals of 6 or more hours on five consecutive days without breaks (Monday–Friday) as described above. Surgery was performed within the following week in 91% of the patients. The median interval from last radiotherapy to surgery was 4 days (3 days: 45%; 4 days: 32%; 5 days: 9%; 6–10 days: 9%; 11–25 days: 5%). In 72% of the patients, a sphincter-sparing resection (SSR) with primary anastomosis was performed, 70% of these were given a temporary protective stoma. In all, 81% of 100 patients receiving surgery at the Department of General Surgery at Vienna University General Hospital had a sphincter sparing procedure, compared to 51 (61%) of 84 patients operated at outside departments of surgery listed in the Acknowledgements (*χ*^2^=4.2; DF=1; *P*<0.05). In 156 patients (85%) a histologically complete resection was achieved, 13% were rated R1, 2% (four patients) were rated R2. As this study did not have a control group without radiotherapy, a comparative assessment of complications during surgery is unavailable. One patient died 8 days after surgery from myocardial infarction. Previously undetected liver metastases had been encountered during surgery in this patient. One other patient acquired a respiratory distress syndrome 9 days after surgery and died on the 43rd postoperative day in the intensive care unit. Thus, 30-day postoperative mortality was 0.5% in this group of patients. The Median stay in hospital after surgery was 13 days.

### Recurrence

The actuarial overall rate (stages I–III) for distant or local recurrence at 4 years was 23% (Table 2). The overall recurrence rate was significantly different between UICC stages I, II, and III. Only three patients suffered a local recurrence at 9, 16, and 18 months. In the last patient, liver metastases had been found at primary surgery and local recurrence occurred during systemic progression, the other two patients had stage II disease with local recurrence as the first site of failure. Thus, the actuarial rate for local recurrence after macroscopically complete resection was 2.1% at 4 years and, remarkably, no further local recurrence has been hitherto encountered in patients with longer follow-up. All local recurrences were presacral within the radiation field. There was no recurrence at the perineum although the whole perineum was not included in the radiation field, even if an APR was planned. In four patients, a macroscopically complete resection was not attained: in one patient the tumour was not resected and a Hartmann situation established, in the other three patients macroscopic tumour was left behind. All of them had T3 or T4 and all had N2 disease and they died at 2, 2, 11, and 14 months, respectively. If these patients are included in the analysis for local control, the actuarial local control rate is 96% at 4 years.

### Survival

**Figure 1:**
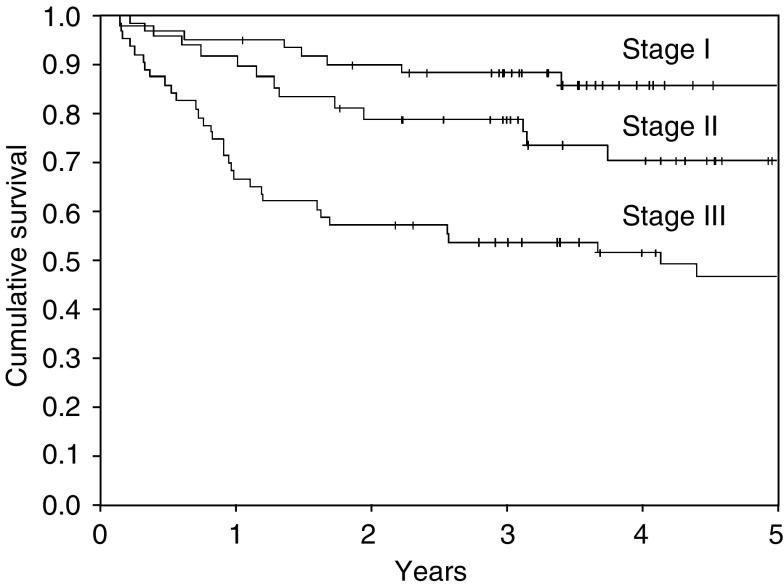
Disease-free survival.

Median follow-up was 53 months for surviving and 43 months for all patients, only five patients had a follow-up of less than 24 months. Actuarial overall and disease-specific survivals for all patients at four years were 68 and 76%, respectively (Table 2). Excluding patients with metastases encountered at surgery, overall survival for patients at stages I–III (*n*=171) at 4 years was 72%, disease-specific survival for these patients was 82%. Disease-free survival was 69% for patients without synchronous distant metastases (Figure 1). Stage III conferred a significantly worse prognosis compared to stages I and II, the difference between stages I and II was not significant. Six of the 184 patients analysed died more than 5 years after treatment at ages 72, 74, 74, 80, 81, and 86. All of them succumbed to intercurrent disease, none had signs of recurrent tumour, and none of these deaths were related to an abdominal condition. The latest metastasis-related deaths occurred at 48 and 56 months, respectively.

### Abdominal complications and adverse effects

In all, 37 patients experienced abdominal or pelvic complications and seven deaths were related to these events (Table 3). All anastomotic stenoses requiring dilation and anastomotic leaks occurred within 3 months post-surgery. Pelvic infections and pelvic bleeding requiring surgical interventions also occurred shortly after cancer surgery. Two rectovaginal fistulae, appearing 7 days and 2 months post-surgery, respectively, were surgically repaired. In all, 70% of patients who underwent primary colorectal or coloanal anastomosis at tumour resection received a protective stoma. Five patients suffered leakage at the site of reconnection after closure of their stoma, three times this was fatal (two ileostomies, one transversostomy). One patient developed an ileus due to stenosis at the site of re-connection, which required surgical revision.

### Bowel function

To assess late bowel function, we report functional results at the last follow-up 5 or more years after treatment for patients treated in 1994–98 (*n*=130). Of these, 77 were alive at final analysis with a median follow-up of 71 months (range 60–123 months). Functional data are available for 68 patients displayed in Table 4. Of these, 22 patients had a well-functioning permanent stoma and were assessed for pelvic pain only, 46 patients with a sphincter in place had a full assessment of bowel function. In all, 78% reported good quality of life. No patient but one reported problems with micturition, this one patient was on chronic dialysis due to intercurrent nonobstructive kidney failure. All patients assessed reported stable bowel function within the year previous to final assessment.

## DISCUSSION

Radiotherapy used as an adjunct to radical surgery reduces the local failure rate from rectal cancer (Camma *et al*, 2000; Colorectal Cancer Collaborative Group, 2001). The 1990 NIH consensus recommends postoperative radiotherapy combined with chemotherapy for patients with UICC stage II and III rectal cancer (NIH consensus conference, 1990). A more recent expert opinion again recommends radiotherapy to be used as an adjunct to surgery preoperatively (short term or long term) or postoperatively (van Cutsem *et al*, 2002). Local failure was reduced in the postoperative radiotherapy arm of the NSABP protocol R-02 (8 *vs* 13% at 5 years), although survival was not affected by radiotherapy (Wolmark *et al*, 2000). In the Swedish Rectal Cancer Trial, the 5-year actuarial local recurrence rate decreased from 27% without to 11% with preoperative short-term radiotherapy of 25 Gy, and survival increased from 48 to 58% at 5 years in the radiotherapy-plus-surgery arm (Swedish rectal cancer trial, 1997). More recently, the local failure rate at 2 years after standardised and quality-assured TME was 8.2%, but only 2.4%, if surgery was preceded by 25 Gy short-term radiotherapy in the Dutch TME trial (Kapiteijn *et al*, 2001).

We present the results of the first 184 consecutively treated patients at our institution from 1994 to 2000, where 25 Gy preoperative radiotherapy was administered using two daily fractions of 2.5 Gy, approaching the conventional fraction size of 2 Gy, but within an overall treatment time of 1 week in a hyperfractionated regimen. The modification was chosen in order to reduce the likelihood of late adverse effects. This assumption is supported by the linear quadratic (LQ) model, the best presently available and widely used model for the estimation of the biologically effective radiation therapy dose (BED) (Glimelius *et al*, 1997; Colorectal Cancer Collaborative Group, 2001). As shown in Table 5b, 25 Gy administered with 2.5 Gy twice daily fractions within 1 week equals a total dose of 34 Gy considering the tumour effect, if it would be delivered conventionally with 2 Gy daily fractions (see also Appendix A). Short-term 5 × 5 Gy daily, the treatment regimen used in Scandinavia, the United Kingdom, the Netherlands, and elsewhere, equals 42 Gy considering the tumour effect. The conventional dose of long-term preoperative radiotherapy for rectal cancer is 45–50 Gy. Considering late normal tissue (adverse) effects, our treatment regimen corresponds to only 28 Gy total dose (assuming an *α*/*β*=3) administered with daily 2 Gy, 5 × 5 Gy corresponds to 40 Gy (other fractionations shown in Table 5b). Given the demonstrated efficacy for preoperative radiotherapy schedules resulting in a BED of ⩾30 Gy_10_ (see Table 5a) in a recent meta-analysis (Colorectal Cancer Collaborative Group, 2001), and given the results described in this paper, 10 × 2.5 Gy administered within 1 week seems to be a treatment regimen with a very favourable risk/benefit ratio (Table 5a and 5b).

In our study, 78% of the patients with a median follow-up of more than 7 years report no impairment of quality of life due to bowel function and 80% are able to delay defecation for more than 15 min upon urge. Only a minority of patients reported a high stool frequency and disturbing clustering. Definitive conclusions cannot be drawn from these data, although they are in agreement with predictions derived from the LQ radiobiological model. It can safely be said that this treatment is very likely to have a low long-term toxicity profile besides its extremely well tolerability in the short run. A definite conclusion regarding late toxicity would require a prospective comparative study, however.

Three patients (2.3% of those with sphincter-sparing surgery) died due to an intestinal leak at the site of reconnection after closure of a protective ileostoma (two patients) or transversostoma (one patient). The latter patient had furthermore developed hepatic failure due to liver cirrhosis shortly after reversal, possibly contributing to perforation. Although this number appears to be relatively high, a relation to radiotherapy could not be found, as the intestine used for the stoma was outside the radiation fields and histology reports of the leaking intestines upon surgical revision revealed no signs of radiation damage.

Swedish researchers have found an increase in bowel movements after preoperative radiotherapy (25 Gy, 5 × 5 Gy) compared to surgery alone, and more patients stated they had an impaired social life due to bowel dysfunction (Dahlberg *et al*, 1998). It is tempting to speculate that modifying the fractionation from 5 × 5 Gy to 10 × 2.5 Gy within the same overall treatment time does reduce the likelihood of functionally relevant adverse late effects in accordance with calculations derived from the LQ model.

Our results provide support that the efficacy of the short-term 25 Gy preoperative regimen is maintained if it is modified to hyperfractionation. By close collaboration between the rectal surgeons and radiotherapists, it is possible to select patients who are sufficiently treated by a nondownstaging short course of preoperative radiotherapy to prevent local failure. This is presently facilitated by using phased-array pelvic MRI (Beets-Tan *et al*, 2001). An actuarial local recurrence rate of 2.1% at 4 years compares favourably with any reported recurrence rates in the literature in comparable patients, especially in a multi-institutional setting with regard to surgery. It needs to be kept in mind that all patients included in this analysis were clinically diagnosed with primarily resectable, locally moderately advanced rectal cancer. Patients with clear clinical or radiological evidence of a small tumour that appeared to be confined to the rectal wall and without suspicious pelvic lymph nodes did not undergo preoperative radiation treatment and are not included in this analysis. The fact that 33% of the surgical specimens were postoperatively diagnosed as stage I (T1: 6%, T2: 27%) reflects the uncertainty of diagnostic modalities used during recruitment to this study (1994–2000) and is in agreement with the Dutch TME trial, where 30% of the patients had stage I disease. As 86% of the patients received surgery within 5 days of the last fraction of radiotherapy, downstaging is unlikely to account for the postoperative rate of stage I tumours. On the other hand, 22% (14 of 63) of T2 tumours had positive lymphnodes, comprising a known risk factor for local recurrence.

Four patients were found unresectable at surgery with T3N2 (two patients, one of whom had liver metastases) and T4N2 (two patients) tumours, an event that is likely to be preventable by the use of pelvic MRI also. Had unresectability been assessed in advance, these patients would not have been included in this study but – at least those without metastases – would have been offered preoperative long-term radiochemotherapy in order to downsize the tumour.

Out of 224 patients operated upon for rectal cancer at the Vienna General Hospital Department of Surgery between 1994 and 2000, 51% did not receive any radiotherapy, 44% received short-term hyperfractionated accelerated radiotherapy to 25 Gy (this study), and 5% received long-term preoperative radiochemotherapy. No patient from this cohort received postoperative radiotherapy within this period. Although the data for chemotherapy were not analysed, all patients were offered 5-fluorouracil-based postoperative chemotherapy at stage II–III if they were medically fit to receive such therapy. Patients with distant metastases received palliative multiagent chemotherapy and were offered surgery for resectable metastases.

Short-term preoperative radiotherapy of 25 Gy administered within 1 week in 10 fractions of 2.5 Gy for resectable localised rectal cancer constitutes a well-tolerated and simple way to increase local control, with long-term adverse effects apparently in a quite tolerable range. This regimen seems to bring about excellent local control without marked late morbidity in primary resectable T3 rectal cancer, where downsizing is not deemed necessary in order to achieve a complete resection. Prospective comparative evaluation of this regimen seems warranted especially in combination with new antineoplastic drugs delivered postoperatively.

## Figures and Tables

**Table 1:** Tumour characteristics

**Postoperative**	***n***	**%**	
*UICC stages*			
Stage I	60	33	
Stage II	48	26	
Stage III	63	34	
Stage IV	13	7	
			
Total	184	100	
			

***pTNM (%)***	**N0**	**N1–2**	**Total**
T1	6	0.5	6.5
T2	27	8 (0.5)^a^	34 (0.5)^a^
T3	26 (0.5)^a^	29 (5)^a^	55 (6)^a^
T4	0.5	3 (0.5)^a^	4 (0.5)^a^
			
Total	59 (0.5)^a^	41 (7)^a^	100 (7)^a^
			

**Surgery**	***n* (%)**	**AKH-DoS**	**Other DoS**
SSR	132 (72)	81 (81)*	51 (61)*
APR	48 (26)	17 (17)	31 (37)
Other	4 (2)	2 (2)	2 (2)
			
	184 (100)	100 (100)	84 (100)

a Numbers in parentheses represent percentage of patients with metastases, encountered at surgery.

* *P*<0.05 (*χ*^2^ test) for SSR, AKH-DoS *vs* other DoS.

SSR: sphincter-sparing resection; APR: abdominoperineal resection; AKH-DoS: Department of Surgery, Vienna Medical University General Hospital; other DoS: Department of Surgery, other institution.

**Table 2:** Survival and overall recurrence rate (numbers are percentages)

	**DSS**		**DFS**		**OS**		**RR**	
	**2y**	**4y**	**2y**	**4y**	**2y**	**4y**	**2y**	**4y**
Stage I (*n*=60)	100	92	90	86	93	84	4	5
Stage II (*n*=48)	98	89	79	70	92	77	15	22*
Stage III (*n*=63)	81	66**	57	52*	73	57*	36	40*
Stage I–III (*n*=171)	92	82	75	69	85	72	19	23
Stage IV (*n*=13)	35	0	NA	NA	35	0	NA	NA
								
All patients (*n*=184)	88	76	NA	NA	82	68	NA	NA

NA: not applicable; DSS: disease-specific survival; DFS: disease-free survival; OS: overall survival; RR: recurrence rate (distant or local).

* *P*<0.05;

** *P*<0.01 calculated by log-rank statistic.

**Table 3:** Abdominal complications

	***n* (%)**	**Of these** **leading to** **death**
Patients with primary anastomosis	132 (100%)	
*Within 3 months post surgery*		
Stenosis requiring dilation	8 (6%)	1 (0.8%)
Anastomotic leakage	15 (11.4%)	3 (2.3%)
Recto-vaginal fistula	2 (1.5%)	0
		
*Later than 3 months post surgery*		
Stenosis requiring dilation	0	0
Anastomotic leakage	0	0
Intestinal leak after closure of stoma	5 (3.8%)	3 (2.3%)
		
All patients	184 (100%)	
Pelvic infection	7 (3.8%)	0
Pelvic bleeding	3 (1.6%)	0
		
Patients with abdominal and/or pelvic complications	37 (20.1%)	7 (3.8%)

**Table 4:** Bowel function at last follow-up 5 years or later (5–10 years), for evaluable patients treated 1994–98 (*n*=68)

	**Good**	**Intermeditate**	**Bad**	**% (*n*)**
Quality of life	78 (36)	20 (9)	2 (1)	100 (46)
Frequency/d	24 (11)	54 (25)	22 (10)	100 (46)
Clustering	26 (12)	61 (28)	13 (6)	100 (46)
Delay upon urge	80 (37)		20 (9)	100 (46)
Distinction stool/gas	78 (36)		22 (10)	100 (46)
Tenesms	96 (43)		4 (2)	100 (46)
Wearing pads	67 (31)		33 (15)	100 (46)
Pelvic pain	100 (68)		0	100 (68)

Numbers represent percentages (*n* in parentheses). Primary anastomosis: 46 patients; permanent stoma: 22 patients.

Categories (from good to bad): quality of life altered by bowel function: no – yes – significantly; stool frequency per day: ⩽1–2–3–>3; clustering: one portion – two to three portions – more than three portions per defecation; ability to delay defecation upon urge: >15 min–<15 min; ability to distinguish between stool and gas: yes–no; experiencing tenesms: no–yes; wearing pads because of fear of incontinence: no–yes; pelvic pain: no–yes.

**Table 5a:** BED using the linear quadratic formula^a^

	**25 Gy (10 × 2.5 Gy)**	**25 Gy (5 × 5 Gy)**	**50 Gy (25 × 2 Gy)**	**45 Gy (25 × 1.8 Gy)**	**50.4 Gy (28 × 1.8 Gy)**
Tumour, *α*/*β*=10 OTT disregarded (Gy_10_)	31.3	37.5	60	53.1	59.5
Tumour, *α*/*β*=10 OTT taken into account (Gy_10_)	31.3	37.5	44.4	37.5	42.1
Normal tissue, *α*/*β*=5 (Gy_5_)	37.5	50	70	61.2	68.5
Normal tissue, *α*/*β*=3 (Gy_3_)	45.8	66.7	83.3	72	80.6

a For calculations see Appendix A.

BED: bioloically effective dose; OTT: overall treatment time (days). For calculations disregarding and taking into account OTT, respectively, see Appendix A.

*α*/*β*: the linear quadratic quotient, set to 10 for tumour effects; set to 5 and 3 for normal tissue effects. Gy_10_ (Gy_5_ and Gy_3_): the biologically effective dose calculated using an *α*/*β* of 10 (5 and 3); Equivalent total dose with 2 Gy daily fractions: the equivalent total dose, if 2 Gy daily fractions would be used, calculated using the linear quadratic formula with *α*/*β* quotients as indicated. Results rounded to multiples of 2 Gy fractions.

**Table 5b:** Equivalent total doses for treatment regimens, for tumour effects and normal tissue effects, if 2 Gy daily fractions would be used^a^

**Equivalent total dose with 2 Gy daily fractionation**	**25 Gy (10 × 2.5 Gy)**	**25 Gy (5 × 5 Gy)**	**50 Gy (25 × 2 Gy)**	**45 Gy (25 × 1.8 Gy)**	**50.4 Gy (28 × 1.8 Gy)**
Tumour, *α*/*β*=10 OTT disregarded (Gy)	26	32	50	44	50
Tumour, *α*/*β*=10 OTT taken into account (Gy)	34	42	50	42	48
Normal tissue, *α*/*β*=5 (Gy)	26	36	50	44	50
Normal tissue, *α*/*β*=3(Gy)	28	40	50	44	48

a For calculations see Appendix A. OTT: overall treatment time (days). For calculations disregarding and taking into account OTT, respectively, see Appendix A.

*α*/*β*: the linear quadratic quotient, set to 10 for tumour effects; set to 5 and 3 for normal tissue effects. Gy_10_ (Gy_5_ and Gy_3_): the biologically effective dose calculated using an *α*/*β* of 10 (5 and 3); Equivalent total dose with 2 Gy daily fractions: the equivalent total dose, if 2 Gy daily fractions would be used, calculated using the linear quadratic formula with *α*/*β* quotients as indicated. Results rounded to multiples of 2 Gy fractions.

## Appendix A

The BED is calculated using the LQ formula. The basic formula takes into account single dose, number of fractions, and the *α*/*β*-quotient: BED=*nd* (1+*d*/*α*/*β*), where *n* is the number of fractions; *d* the dose (Gy) per fraction; *α*/*β* the LQ quotient. The LQ quotient is usually set to 10 for acute (tumour) effects, and to 3 or 5 for late (normal tissue) effects. For late effects in normal tissue, the impact of overall treatment time is generally not regarded relevant (Jones and Dale, 1999). For tumour effects, the overall treatment time is taken into account by incorporating the repair rate *γ*/*α*, set to 0.6 Gy per day, which is a measure for how much dose is lost per day due to tumour tissue repair. The proliferation delay *T*_*k*_, set to 7 days (Colorectal Cancer Collaborative Group, 2001), is subtracted from the overall treatment time *T*, which means that, after a delay of 7 days, repair mechanisms become relevant that lead to a loss of tumour-damaging effects at a magnitude of 0.6 Gy per day. Thus, the formula for tumour effects taking into account the overall treatment time (including weekends) is

with an *α*/*β* of 10. If the overall treatment time is shorter than the proliferation delay *T*_*k*_, the term would be negative and has therefore to be set to 0 in these cases (the case for 5-day overall treatment time). As repair rate and proliferation delay carry a higher degree of uncertainty than the *α*/*β* quotient, results are also reported for BEDs disregarding the effects of overall treatment time in Tables 5a and 5b. It can be seen that in shorter treatment regimens (5 days overall treatment time) the respective effect of 1 Gy administered is higher for tumour than for normal tissue, because a much smaller total dose achieves an effect upon the tumour that would require a higher total dose, if administered over a longer overall treatment time (Table 5b). Conventional radiotherapy treatments are administered with daily fractions of 2 Gy. To understand the biological tumour and normal tissue effects of unconventional fractionations such as 5 × 5 Gy or 10 × 2.5 Gy with two fractions administered daily, it is helpful to calculate total doses delivered with conventional fractionation, that would be biologically equivalent with the regimen in question. This is done by seeking the number of fractions of 2 Gy necessary to yield the BED of the regimen in question (e.g. 10 × 2.5 Gy), of course using the same parameters for the *α*/*β* quotient. In this way, equivalent total doses for different *α*/*β* quotients are obtained that can be used to estimate the tumour and normal tissue effects of an unconventional treatment regimen (Table 5b).
